# Validation of inner ear MRI in patients with Ménière’s disease by comparing endolymphatic hydrops from histopathologic specimens

**DOI:** 10.1038/s41598-021-97213-7

**Published:** 2021-09-06

**Authors:** Young Sang Cho, Jong Sei Kim, Min Bum Kim, Sung Min Koh, Chang Hee Lee, Yi-Kyung Kim, Hyung-Jin Kim, Won-Ho Chung

**Affiliations:** 1 Department of Otorhinolaryngology-Head and Neck Surgery, Samsung Medical Center, Sungkyunkwan University School of Medicine, 81 Irwon-ro, Gangnam-gu, Seoul, 06351 South Korea; 2 Department of Radiology, Samsung Medical Center, Sungkyunkwan University School of Medicine, Seoul, South Korea

**Keywords:** Brain, Magnetic resonance imaging

## Abstract

Intravenous gadolinium-enhanced inner-ear magnetic resonance imaging (IV-Gd inner-ear MRI) has been used to visualize endolymphatic hydrops (EH) in clinical diagnosis of Ménière’s disease (MD). However, lack of histological validation has led to several concerns regarding how best to interpret the resulting images. Here, we compared hydropic changes in temporal bone specimens with the results of IV-Gd inner-ear MRI in patients with MD. Histopathologic images of temporal bones from 37 patients with MD and 10 healthy controls were collected from the National Temporal Bone Bank of the Massachusetts Eye and Ear Infirmary in the United States. The EH ratios in the vestibule and cochlea were calculated from temporal bones using the methods used for IV-Gd inner-ear MRI, and the degree to which the saccular and utricular hydrops contributed to vestibular hydrops was measured. The presence of hydropic change in each semicircular canal was assessed using temporal bone images and compared with IV-Gd inner-ear MRI scans of 74 patients with MD. Based on human temporal bone imagery, the EH ratios in the cochlea and the vestibule on the affected side were 0.314 and 0.757, respectively. In the healthy control group, the ratio was 0.064 for the cochlea and 0.289 for the vestibule; these values were significantly different from those for the affected side of MD patients. The values for the affected ear were similar to the ratios from the IV-Gd inner-ear MRI scans in MD patients. In the vestibule, saccular hydrops were more common than utricular hydrops. The average EH ratios in the saccule and utricle were 0.513 and 0.242, respectively. No significant hydropic change from each of three semicircular canals was evident in temporal bone histopathology. However, herniation of otolithic organs (saccule or utricle) into the lateral semicircular canal was found in 44.4% of the patients, with saccular herniation (24.8%) more common than utricular herniation (16.7%). Although IV-Gd inner-ear MRI might not reflect fully the results of actual histopathology due to the limited resolution of MRI and image-processing techniques, the measured EH ratios from temporal bone specimens and IV-Gd inner-ear MRI scans were similar. Hydropic change in the three semicircular canals was not significant at either the ampullated or nonampullated end. Canal invasion of vestibular hydrops seen on MRI also appeared in temporal bone histopathology, and saccular invasion was dominant.

## Introduction

In 1861, Prosper Ménière described fluctuations in hearing loss and episodic vertigo as evidence of dysfunction of the inner labyrinth rather than a central neurogenic disorder^1^. In 1938, Yamakawa^2^ and Hallpike and Cairns^3^ identified endolymphatic hydrops (EH) as a histopathological marker in patients with Ménière’s disease (MD)^4–6^.

The diagnosis of MD is based on criteria proposed by the American Association of Otolaryngology–Head and Neck Surgery^7^ and the Bárány Society^8^. These diagnostic criteria were based on clinical manifestations because of a lack of clear and objective measures to confirm MD.

Recently, magnetic resonance imaging (MRI) has been reported to be a useful tool for diagnosing MD in most patients through in vivo visualization of EH^9^. It also can be used to differentiate MD from other diseases^10,11^. Correlations between audiovestibular tests and hydrops level have been reported^12,13^. Pure-tone audiometry and electrocochleography (ECoG) have been correlated with severity of hydrops in the cochlea and vestibule^14^. In contrast, the correlation of caloric function and vestibular evoked myogenic potential (VEMP) with hydrops level is controversial^15^. In addition, changes in hydrops level after treatment or over time have been reported^16^. The successful use of MRI to visualize EH and assess the hydrops level in the inner ear would provide valuable information regarding diagnosis, treatment options, and treatment outcomes in patients with MD. Optimal methods to obtain and validate reliable data are required.

However, because image acquisition and processing and data interpretation differ among study groups, concerns regarding the technique’s accuracy persist. Image quality can differ according to the delivery method for gadolinium (intravenous vs. intratympanic) and MRI parameters such as inversion time^17^. In addition, the method used to measure hydrops level might not be accurate. MRI data must be validated by comparisons with histopathologic findings from the temporal bones of patients with MD.

In previous studies, we reported that intravenous gadolinium-enhanced inner-ear MRI (IV-Gd inner-ear MRI) was useful for diagnosis of definite MD. Hydrops level was correlated with pure-tone thresholds, cochlear summating potential/auditory nerve action potential on ECoG, and caloric tests. However, it was not correlated with VEMP thresholds^13^. We also documented that discrepancies between caloric tests and video head impulse test (vHIT) results were the product of hydropic change in the horizontal canal rather than actual vestibular loss. Recently, deep-learning techniques using artificial intelligence have been used to measure automatically the hydrops ratio^18^. Advances in image acquisition and measuring techniques might be applicable. However, several issues have to be clarified with respect to histologic findings.

This study was designed to identify histopathologic features required to address successfully several controversial issues raised by previous MRI studies. First, we compare our methods of measuring hydrops ratio in the cochlea and vestibule. Second, we address the controversy surrounding the correlation of VEMP and MRI. Assuming different contributions from saccular and utricular hydrops in vestibular hydrops, we measured saccular and utricular hydrops levels separately to assess the contributions of saccules and utricles in vestibular hydrops. Third, to confirm the existence of hydrops change in each semicircular canal, the hydrops level at the ampullated and nonampullated ends of each semicircular canal was measured. Fourth, because the correlational of caloric tests and MRI has been controversial, we investigated hydropic changes and canal invasion of vestibular hydrops. Our results help to elucidate these controversial issues and provide the groundwork for further studies.

## Results

### EH ratios for the cochlea and vestibule in temporal bone histopathology and IV-Gd inner-ear MRI

**Figure 1:**
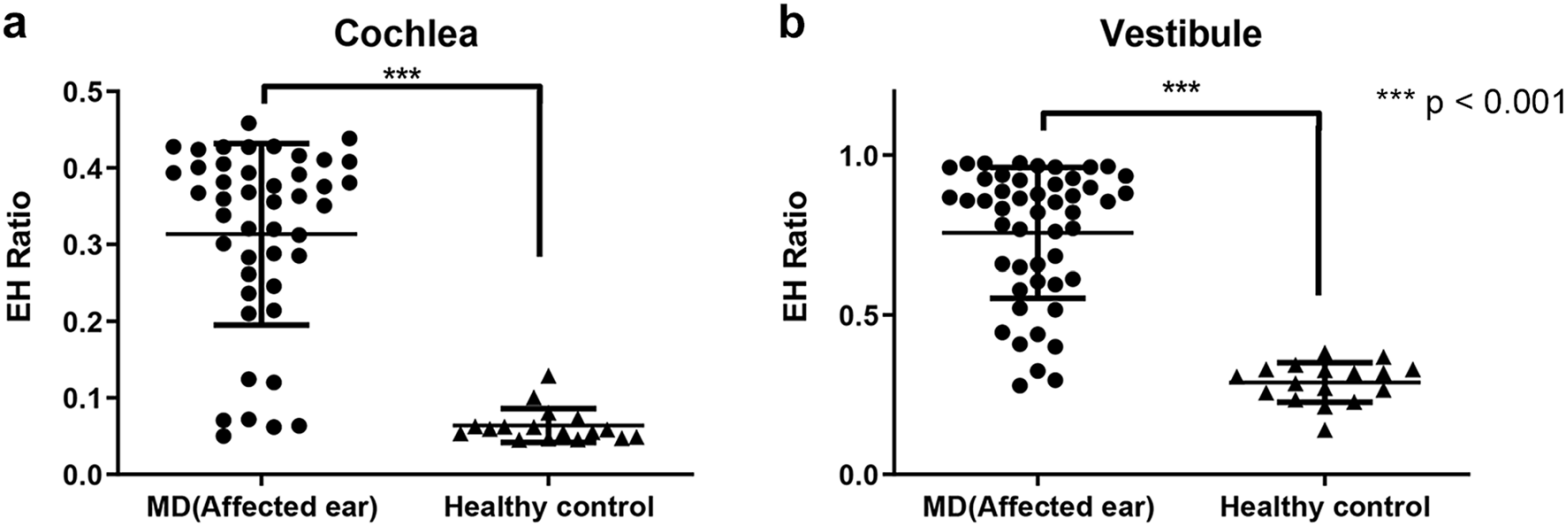
Endolymphatic hydrops (EH) ratios analyzed from histopathology images. In both cochlea (**a**) and vestibule (**b**), the EH ratio of the affected ear was significantly higher (p < 0.001) than that of the healthy control group.

Ratios for EH from temporal bone specimens in 37 MD patients and 10 healthy control patients were measured in the cochlea and vestibule (Fig. 1). The average EH ratio [standard deviation (SD)] in the cochlea was 0.314 (0.118) on the affected side (54 ears) and 0.064 (0.022) in the healthy control group. In the vestibule, the average EH ratio was 0.757 (0.205) and 0.289 (0.062) in the affected side and healthy controls, respectively. In both cochleas and vestibules, the affected ears showed a significantly higher EH ratio value (p < 0.001) compared with healthy controls.

**Figure 2:**
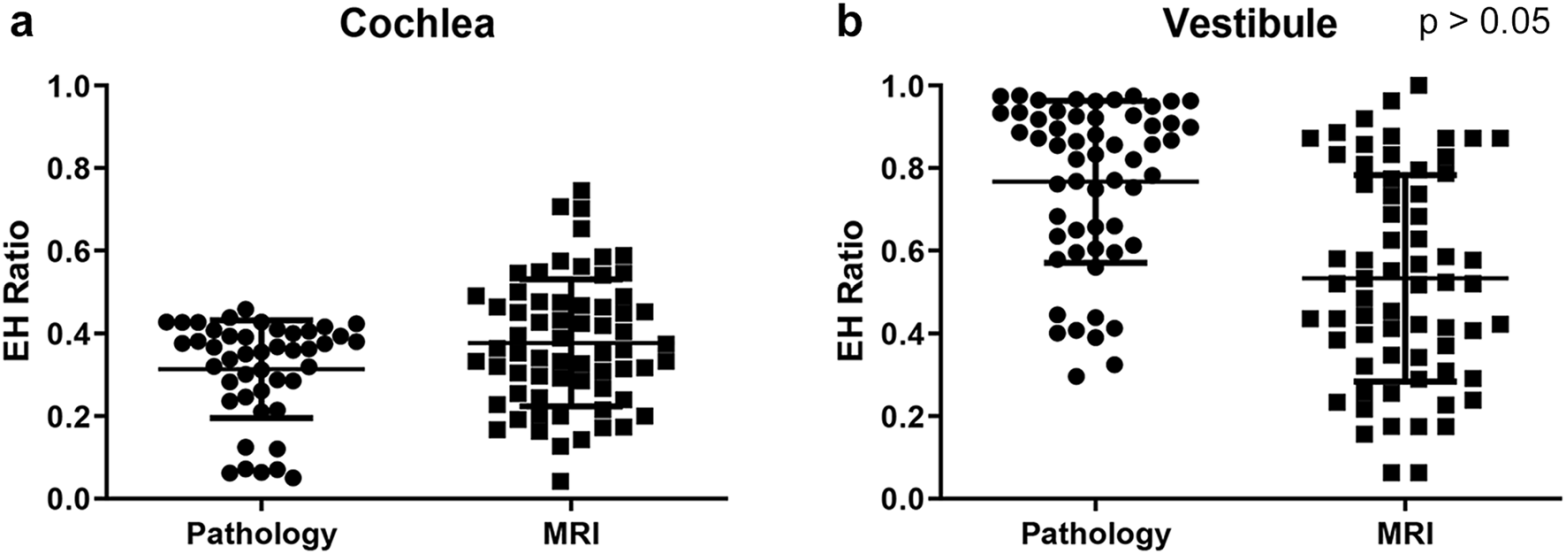
Comparison of histopathology and MRI endolymphatic hydrops (EH) ratios in affected ears. The mean values for the EH of affected ears in the cochlea (**a**) and vestibule (**b**) were not significantly different between histopathology and MRI.

These values were compared with the EH ratio calculated from inner-ear MRI of 72 patients with unilateral definite MD. The mean hydrops ratio in the cochlea from inner ear MRI was 0.372 (0.164), which was similar to the mean hydrops ratio calculated from histopathology (mean, 0.314) (Fig. 2a). In addition, the mean hydrops ratio in the vestibule was not significantly different between histopathology (0.757) and MRI (0.533; SD, 0.250) (Fig. 2b). Therefore, the mean hydrops ratio in the cochlea and vestibule was comparable between histopathology and inner ear MRI using our methods.

### Contributions of saccular and utricular hydrops to vestibular hydrops

**Figure 3:**
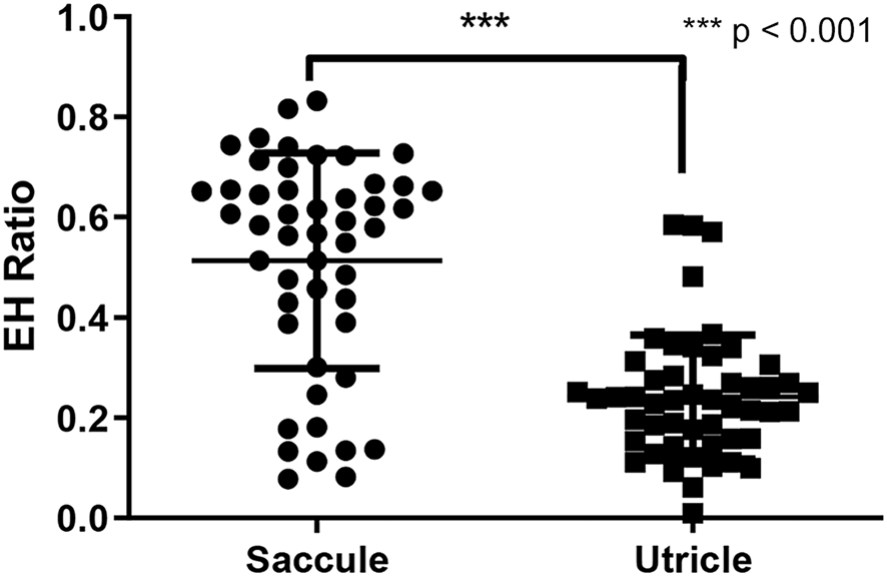
The ratio of endolymphatic hydrops (EH) measured in the saccule and utricle of the vestibule on histopathology.

From IV-Gd inner-ear MRI, the mean hydrops ratio (SD) in the affected vestibule was 0.533 (0.250). However, it was difficult to separate saccular and utricular hydrops in the MR images using our method. We therefore evaluated saccular and utricular hydrops separately using temporal bone specimens. Using specimens from the affected side, the mean numbers (SD) of saccular and utricular hydrops were 0.513 (0.214) and 0.242 (0.124), respectively. The saccular hydrops ratio was significantly higher (p < 0.001) than the utricular hydrops ratio (Fig. 3).

### EH ratios in semicircular canals in temporal bone histopathology

**Figure 4:**
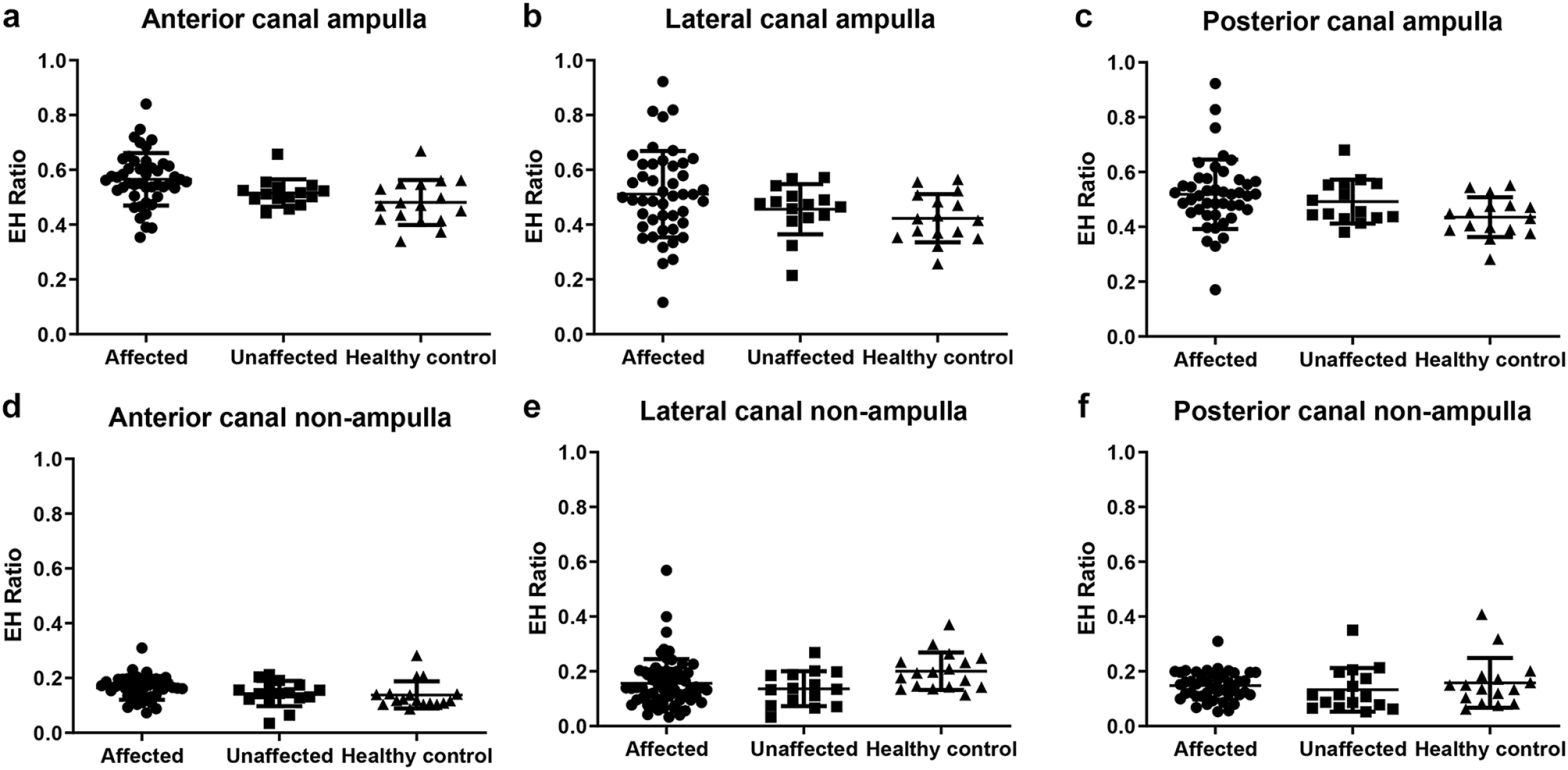
The endolymphatic hydrops (EH) ratio in all semicircular canals analyzed from histopathology images. All canals were analyzed by dividing them into an ampulla end (**a**–**c**) and a non-ampulla end (**d**–**f**). No statistical differences were evident between them.

The EH ratios in three semicircular canals were measured at either the ampullary or non-ampullary region. For the affected ear, the mean endolymphatic space ratios (SD) in the ampullary region of anterior, lateral, and posterior canals were 0.565 (0.096), 0.511 (0.158), and 0.518 (0.068), respectively. In the unaffected ear, the EH ratios of the anterior, lateral, and posterior canals were measured to be 0.515 (0.050), 0.456 (0.092), and 0.491 (0.080), respectively, compared with the affected side. Overall, no significant differences were found between the affected, unaffected, and healthy control groups (Fig. 4a–c). The same results were obtained from non-ampullary regions of each semicircular canal (Fig. 4d–f). We concluded that hydropic change was not significant in any semicircular canal in MD.

### Canal extension of EH from the vestibule in temporal bone histopathology and IV-Gd inner-ear MRI

**Figure 5:**
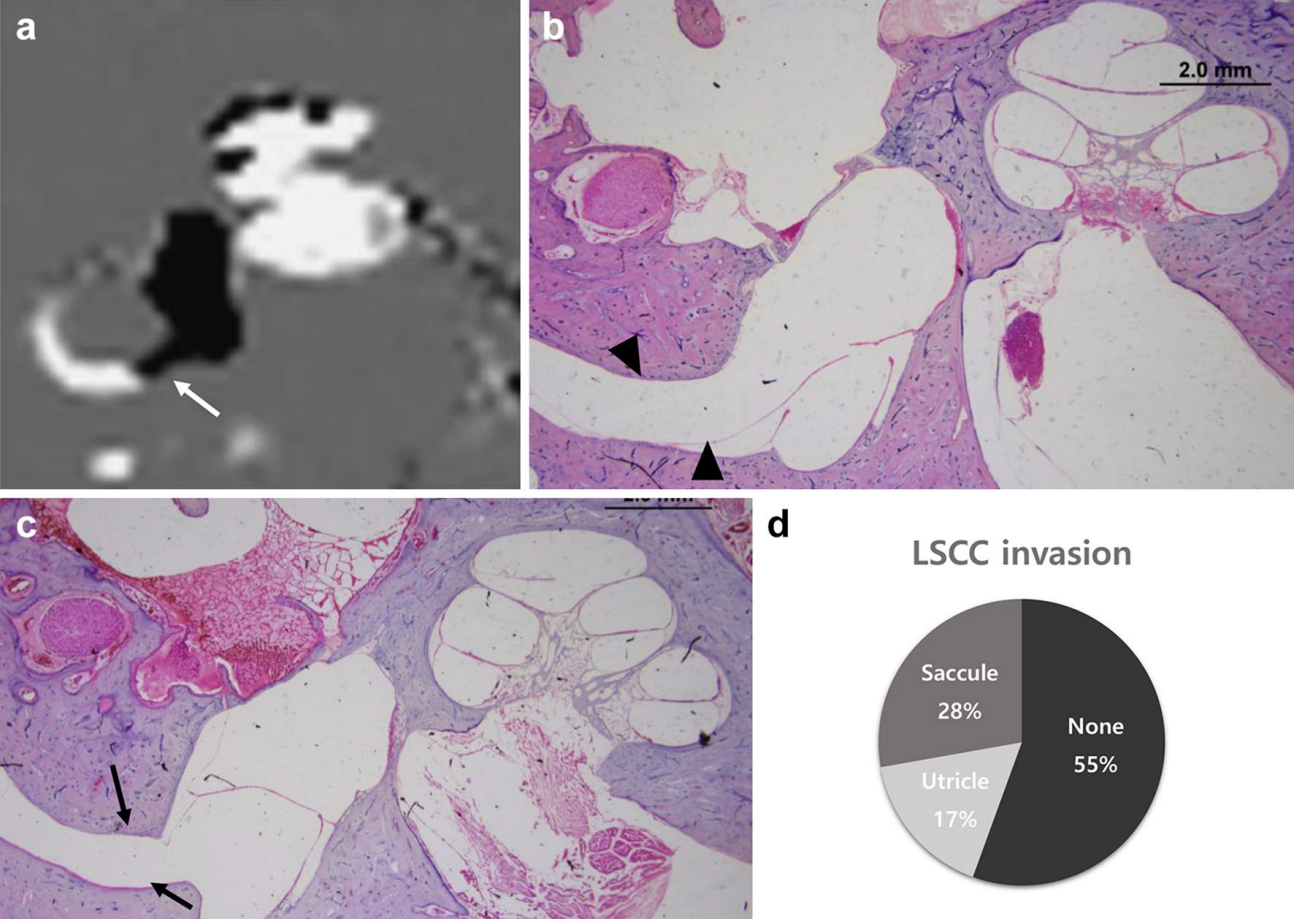
Vestibule endolymphatic hydrops (EH) observed on MRI and histopathology images. On inner-ear MRI, the hydrops of the vestibule extended toward the non-ampullary region (white arrow) of the lateral semicircular canal (LSCC) (**a**). In the histopathological section, the saccular hydrops (black arrowhead) progressed and extended to the LSCC, (**b**) and the utricle also invaded the LSCC (black arrow) (**c**). The rate of extension of vestibular hydrops to the LSCC in histopathological images of MD patients (**d**, 72 ears). *(**a**) image was generated by OsiriX MD image software(version 7.5.1 64-bit, https://www.osirix-viewer.com).

In IV-Gd inner-ear MRI, canal extension of EH from the vestibule was found in 29 cases (40.3%), as shown in Fig. 5a. It was difficult to determine in the MRI scans whether this was due to extension of the utricle or the saccule. However, in temporal bone specimens, they could be distinguished clearly, as shown in Fig. 5b,c (saccule and utricle, respectively). In temporal bone specimens, a canal extension from the vestibule was observed in 24 (44.4%) of the 54 affected ears. Among them, saccular hydrops (n = 15) was observed more commonly to extend into the lateral semicircular canal (LSCC) compared with utricular hydrops (n = 9) (Fig. 5d). There was no case in which the saccule or utricle extended to the LSCC in the healthy control group.

## Discussion

This study was intended to validate data from IV-Gd inner-ear MRI visualizations of the EH of the cochlea and vestibule by comparing them with histopathologic findings in human temporal bone specimens. Hydropic changes in the endolymphatic space of the semicircular canal and canal invasion of vestibular hydrops were investigated through histopathologic review.

Because EH has been known as a histopathological marker since the 1930s^19,20^, histopathologic analysis has long been applied to MD patients. However, the pathologic mechanisms underlying the development of EH are not clear^21^. Gadolinium-enhanced inner-ear MRI was developed to visualize endolymphatic hydropic change in the inner ears of patients with MD, and multiple lines of research are possible regarding the pathogenesis of MD and other inner-ear diseases related to hydropic change^22^.

Methods for visualizing EH by inner ear MRI include calculating the endolymphatic hydrops ratio or the ratio of saccule to utricle. The method used to measure hydrops ratio in the vestibule was introduced by Naganawa et al.^23^, and it could not assess easily the contributions of saccule and utricle in vestibular hydrops^24^. In contrast, the saccule to utricle ratio inversion (SURI) method can be useful for measuring saccular hydrops in early hydrops; however, it is difficult to calculate the saccule to utricle ratio in severe hydrops^25,26^.

Two delivery methods for Gd have been proposed: intratympanic and intravenous. Each method presents advantages and disadvantages, including patient compliance, waiting time, and perilymph signal-to-noise ratio. In addition, because MRI acquisition techniques and qualitative and quantitative methods for analyzing hydrops differ among research teams, the resulting data are inconsistent. We therefore needed to validate MRI data by comparing them with histopathologic findings from temporal bone specimens of MD patients. Both IV-Gd inner-ear MRI and quantitative analysis as described by Nagakawa et al.^23^ were used for this study.

### Comparison of hydrops ratio in the cochlea and vestibule in temporal bone specimens and IV-Gd inner-ear MRI

Inner-ear MRI is used widely to visualize EH in the cochlea and vestibule of patients with definite MD. Between 90 and 100% of MD patients reportedly exhibit prominent EH in the cochlea and/or vestibule according to MRI scans^24,27,28^. This frequency is similar to that associated with histopathological studies^29^. In the histopathology specimens used in the present study, the EH ratio of the affected side averaged 0.314 for the cochlea and 0.757 for the vestibule. This ratio is similar to that observed in actual IV-Gd inner-ear MRI (cochlea, 0.372; vestibule, 0.533). Although perfect comparisons are impossible because the subjects of the MRI and histopathology specimens were different, the hydrops ratio obtained from the IV-Gd inner-ear MRI was comparable to that of real temporal bone specimens. As a result of MRI analysis, some of the EH ratios were less than 0.2, but this was a result of the presence of hydrops in only the cochlea or vestibule alone, not both. In all affected ears, the EH ratio of either cochlea or vestibule exceeded 0.3 in MRI, and results of histopathological reviews were similar.

### Role of saccular and utricular hydrops in EH in the vestibule: implications for VEMP correlation

Although MRI techniques have advanced in recent years, accurate evaluation of EH remains elusive. For example, in the case of severe hydrops shown in Fig. 5a, it is difficult to distinguish between the saccule and utricle in the vestibule using our method. The correlation between VEMP and vestibular hydrops is controversial. Gurkov pointed out that these results were due to the inhomogeneity of diagnostic criteria, hydrops quantification, and VEMP quantification^22^. The cVEMP test is commonly used to assess saccular dysfunction. Several studies have reported that the sensitivity of cVEMP in MD patients ranges from 50 to 70%^30–32^. As our results showed, VEMP correlation might be dependent on the involvement of otolithic organs. In temporal bone specimens, vestibular hydrops detected by MRI was influenced by either saccular or utricular hydrops, with saccular hydrops the more common of the two. Therefore, VEMP results can be affected by involvement of otolithic organs and severity.

### Hydrops changes in semicircular canals and hydropic extension into semicircular canals: Implications for caloric test correlations

McGarvie et al. described a mechanism to explain the decrease in caloric response and discrepancy of vHIT results^33^. According to their theory, hydropic expansion of the endolymphatic duct in MD patients increases turbulence within the duct, and this can dissipate the hydrostatic pressure caused by thermally induced density differences and diminish or eliminate deflection of the cupula. In our results, no significant dilation of semicircular canal endolymphatic ducts was observed on the affected sides in MD patients, even though there were several outliers at the ampullated end (Fig. 4). However, the saccule or utricle was enlarged and invasion into the non-ampulla end of LSCC was seen in 45% of cases (Fig. 5d). Saccular hydropic extension into the horizontal canal has been reported frequently, but utricular hydropic extension is rare in histopathological analysis^34^. In our analysis, utricular invasion was observed in 17% of patients, which led us to speculate that the deterioration of caloric response in MD patients was due to vestibule invasion, not dilatation of the endolymphatic duct within the canal. In a previous study using IV-Gd inner-ear MRI^13^, significant caloric response degradation (canal paresis) was seen only in these groups and not in those without canal invasion, which supports our findings and explains the recently reported dissociation of caloric and vHIT results in patients with MD or delayed EH^35,36^.

Our research has several limitations. The temporal bone specimens were not from the same patients subjected to IV-Gd inner-ear MRI, prohibiting direct histological validation. The subjects who supplied the temporal bone specimens were older and more likely to have end-stage MD without active vertigo spells compared with the subjects studied by MRI who had likely active MD. In addition, the severity of hydrops might differ between the two groups. Despite these limitations, our results showed similar hydrops ratios between the two groups, and the contributions of the saccule and utricle to vestibular hydrops and canal invation were identified. Additionally, several controversial issues regarding VEMP and caloric result correlations were explained. In this study, we validated MRI findings of hydrops ratio and canal extension by comparing them to histopathologic findings and demonstrated controversial issues such as VEMP and caloric test correlation.

## Conclusions

Although we did not make direct comparisons between inner ear MRI and histopathology from the same patients, our comparisons showed that hydrops ratios measured using inner ear MRI were similar to those obtained through histopathology. In addition, hydropic change in the semicircular canal was not significant at either the ampullated or non-ampullated end, and the canal typically was invaded by vestibular hydropic extension, mainly by the saccule. Due to the limited resolution of MRI and the need for additional image processing, we were unable to fully duplicate the results of actual histopathology. However, MRI is promising for study of MD patients.

## Materials and methods

### Subject enrollment for MRI

Data from 72 MD patients (33 males, 39 females; mean age = 49.9 years, age range = 19–75 years) were evaluated in this study. All patients were diagnosed with definite MD according to the Committee of the Bárány Society diagnostic criteria^8^. Seventy-one patients had unilateral MD. The average symptom duration was 51.9 months (range = 1.3–281.9 mo), and there were no patients with history of any other otologic disease or surgery. The pure tone average (0.5K, 1K, 2K, 4K) at the lesion side was 50.19 dB HL (SD = 19.58 dB). Written informed consent was obtained from all participants prior to conducting the study. This study was approved by the Institutional Review Board of Samsung Medical Center following the tenets of the Declaration of Helsinki (IRB File No. 2018-11-020).

### MRI protocol

The protocol described below is the same as that reported by Naganawa et al. in 2012^37^. IV-Gd inner-ear MRI was performed on a 3.0-T unit (MAGNETOM Skyra; Siemens Medical Solutions, Erlangen, Germany) using a 32-channel array head coil. All patients waited 4 h after a single dose (0.1 mL/kg or 0.1 mmol/kg body weight) of IV-administered gadobutrol (gadolinium-DO3A-butriol, GADOVIST 1.0; Schering, Berlin, Germany) before undergoing MRI. All patients underwent heavily T2-weighted (hT2W) magnetic resonance cisternography (MRC) for anatomical reference of total endolymphatic fluid, hT2W–3D-FLAIR with an inversion time of 2250 ms [positive perilymph image (PPI)], and hT2W–3D-IR with an inversion time of 2050 ms [positive endolymph image (PEI)] for evaluating EH. Repetition time was 9000 ms, echo time was 540 ms, and voxel size was 0.5 × 0.5 × 1.0 mm.

The PEI parameters were the same as those for PPI, with the exception that PEI had an inversion time of 2050 ms. The MRC, PPI, and PEI employed identical fields of view, matrix sizes, and slice thicknesses to facilitate comparisons. We produced HYDROPS images on the scanner console by subtracting the PEI from the PPI. To increase the contrast-to-noise ratio of the HYDROPS images, HYDROPS-Mi2 images were generated on a DICOM viewer (OsiriX MD image software, version 7.5.1 64-bit; Pixmeo Sarl, Bernex, Switzerland, https://www.osirix-viewer.com) by multiplying the HYDROPS and MRC images^23^.

All patients underwent pure-tone audiography (PTA) at 6 frequencies (0.25, 0.5, 1.0, 2.0, 4.0, and 8.0 kHz). A semi-automated testing device was used in a sound-attenuating booth that met the prevailing standards for maximum permissible ambient noise levels during audiometry (ANSI, 1977).

### Data annotation from MRI

One neuro-radiologist and one neuro-otologist independently evaluated the MRI scans. According to methods proposed by Naganawa et al.^23^, each physician manually drew a contour of the cochlea and vestibule on the MRC image. The region of interest (ROI) was established as follows. 1. First, the image window level and width were altered to 400 and 1000 pixels, respectively, to obtain optimal visual clarity. (2) For the cochlea ROI, the slice visualizing the cochlea turns (basal, middle, and apical) was selected. If every turn was visible on 2 or more slices, the slice with the greatest height of the modiolus was chosen as a representative cochlea slice. (3) For the vestibular ROI, the lowest slice in which the LSCC ring was visible for more than 240º was selected, and the ampulla was excluded on MRC images. The ROIs drawn on IV-Gd inner-ear MRI were copied and pasted onto HYDROPS-Mi2 images. The histogram function in the OsiriX program was used to estimate the numbers of pixels in the ROI and with negative signal intensity values (i.e., endolymph) in the ROI. The EH ratio was calculated manually as the number of pixels for the endolymph in the ROI divided by the total number of pixels in the ROI.

### Human temporal bone specimens

Temporal bone histopathologic specimens were collected from the National Temporal Bone Database in Massachusetts Eye and Ear Infirmary in the United States. A keyword search of the database using the term “Ménière” produced 105 cases (May 2019). Among them, patients who were diagnosed as non-MD in their lifetime and those who underwent a labyrinthectomy, decompression or shunt, cochlear implant, stapedotomy, or stapedectomy were excluded. In addition, patients were excluded when their slides were difficult to read due to artifacts or moderate-to-severe post-mortem autolysis. In total, 37 MD cases (72 ears) were included in the analysis (18 males, 19 females). After histologic review of 37 patients, 13 were found to have bilateral endolymphatic hydrops and were diagnosed with bilateral MD. The average of the four frequencies on the lesion side was 63.94 dB HL (SD = 22.14 dB), but accurate evaluation of symptom duration was not possible because many records were missing. Patient history and PTA results were reviewed in the healthy control group, and 10 patients (17 ears, 4 males, 6 females) with no hearing loss and no history of vertigo were analyzed. Their average pure tone hearing was 40.44 dB HL (SD = 27.59). All specimens were prepared for light microscopy by fixation in 10% buffered formalin (or Heidenhain’s Susa solution), decalcification in ethylene diamine tetra-acetic acid or trichloroacetic acid, and dehydration in graded alcohols. The samples were then embedded in celloidin and sectioned at a thickness of 20 mm through the axial plane. Every 10th section was stained with hematoxylin and eosin and mounted on glass slides.

### Data annotation from temporal bone specimen

**Figure 6:**
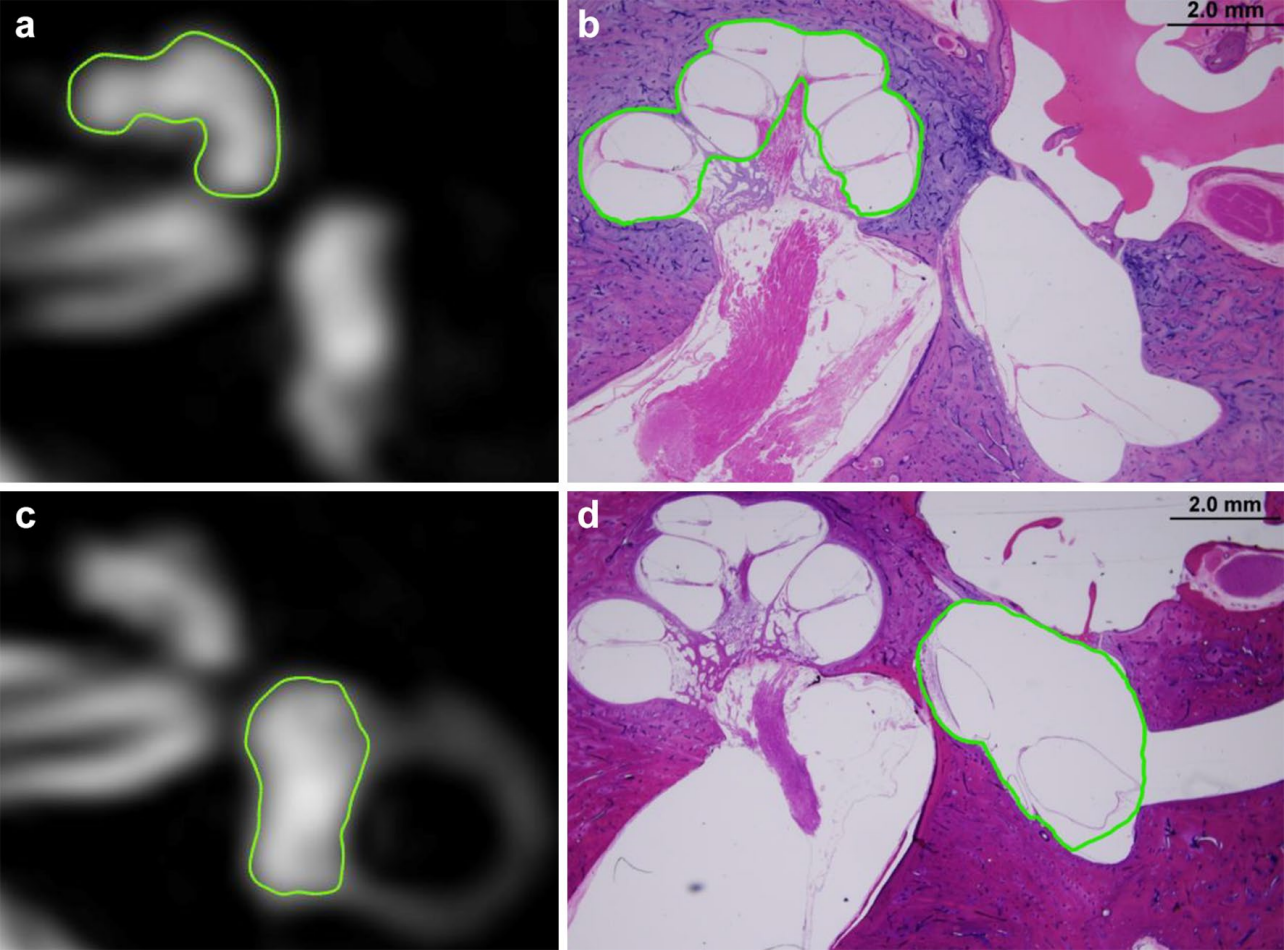
In the MRC image of the inner ear MRI and histopathology image, the region of interest for each organ is indicated by the cochlea (**a**,**b**) and vestibule (**c**,**d**), respectively. *(**a**) and (**c**) image was generated by OsiriX MD image software (version 7.5.1 64-bit, https://www.osirix-viewer.com).

To measure the endolymphatic space of cochlear, vestibule, and semicircular canals, we used ImageJ software (http://rsb.info.nih.gov/ij), which is freely available and the most widely used scientific image analysis program^38^. Representative slices of the cochlea and vestibule were selected in the same manner as for MRI analysis, and images were obtained of the representative slide magnified 200 × with an optical microscope. One neuro-otologist manually drew a contour of the cochlea and vestibule on the pathology slide image using ImageJ. The cochlea was divided into basal, middle, and apical turns, and the boundaries of the scala media (the space between the Reissner’s membrane and the basilar membrane, endolymphatic space) of each turn were drawn. After adding this endolymphatic space area, the EH ratio was obtained by dividing it by the whole cochlea area. The software also draws the contours of utricle and saccule from the representative image slide and divides them into the entire vestibule area (Fig. 6). The semicircular canals were measured by dividing the canal’s cross-sectional area by the area of the endolymphatic space in each of the ampullary and non-ampullary regions.

### Statistical analysis

We used SPSS 18.0 software (SPSS Inc., Chicago, IL, USA) for all statistical analyses and adopted a p-value < 0.05 as the statistical significance threshold. To compare the average of the EH ratios between groups, an independent sample *t* test or Mann–Whitney test was performed after the normality test.
